# Surgery for Trapeziometacarpal Joint Arthritis: A Network Meta-Analysis of Randomized Studies

**DOI:** 10.1016/j.jhsg.2025.100737

**Published:** 2025-05-22

**Authors:** Dimitris Challoumas, Ali Hamad, Varun Rana, Amit Putti, Neal L. Millar

**Affiliations:** ∗ Institute of Infection and Immunity, University of Glasgow, Glasgow, UK; † Department of Trauma & Orthopaedics, Forth Valley Royal Hospital, Larbert, UK; ‡ School of Medicine, University of Glasgow, Glasgow, UK; § School of Medicine, University of Dundee, Dundee, UK

**Keywords:** Osteoarthritis, Prosthesis, Replacement, Suspensionplasty, Trapeziectomy

## Abstract

**Purpose:**

Our aim was to compare the efficacy and safety of the available surgical interventions for trapeziometacarpal joint (TMCJ) arthritis.

**Methods:**

We conducted a systematic review and network meta-analysis of randomized studies comparing surgical interventions for TMCJ arthritis. Our primary outcome was patient-reported pain, and secondary outcomes were patient-reported functional disability, key pinch strength, and complications. Mean differences (MD) and standardized mean differences (SMD) were calculated for continuous outcomes and odds ratios (OR) for dichotomous outcomes, all with 95% confidence intervals.

**Results:**

Twenty-one randomized studies were included. In pairwise meta-analyses, when simple trapeziectomy was compared with trapeziectomy with ligament reconstruction and tendon interposition (LRTI), all pain, functional disability, and key pinch strength were similar (pain visual analogue scale, MD 0.22 points [−0.66 to 0.21]; functional disability, SMD 0.17 [−0.06 to 0.41]; key pinch strength, MD 0.07 kg [−0.22 to 036], *P* = .64). Compared with trapeziectomy (with or without LRTI), total joint arthroplasty provided similar short-term pain relief (MD 0.20 points [−0.10 to 0.50], moderate certainty of evidence), statistically greater improvement in short-term functional disability (disabilities of the arm, shoulder, and hand), and key pinch strength (function disabilities of the arm, shoulder, and hand, MD 5.24 points [0.72–9.75], low certainty of evidence; key pinch strength, MD 0.92 kg [0.63,–1.21], and moderate certainty of evidence]). Only the difference in key pinch strength exceeded clinical significance. In network meta-analyses, total joint arthroplasty ranked first for all short-term pain, function, and key pinch strength; however, its superiority over other treatments was only significant for key pinch strength. Short-term complications between simple trapeziectomy and trapeziectomy with LRTI, and between trapeziectomy and total joint arthroplasty were similar; however, longer-term data were not available.

**Conclusions:**

Total joint arthroplasty appears to be at least as effective as trapeziectomy for pain relief and functional disability and may be superior for key pinch strength in the short-term. Until long-term efficacy, complication, and survivorship data arising from high-quality comparative studies become available, its widespread use cannot be recommended.

**Type of study/level of evidence:**

Therapeutic II.

The trapeziometacarpal joint (TMCJ) is a bi-concave saddle joint that is prone to degeneration because of its high range of motion.^1^ TMCJ arthritis is more prevalent with increasing age, particularly in postmenopausal women. Excessive basal joint laxity, which is common in young women, may predispose to the condition as a result of repeated loading of the subluxated joint.^1^^,^^2^ Approximately one-third of people over the age of 50 have been found to have radiographic osteoarthritis of the TMCJ in epidemiological studies, increasing up to 91% in the 80s; however, this has a poor correlation with clinical symptomatology.^3^ The main clinical features are pain and impaired hand function, especially reduced pinch and grip strength.

First-line treatment for patients with mild symptoms includes activity modification, nonsteroidal anti-inflammatory drugs, splinting, strengthening exercises, and intra-articular injections.^4^^,^^5^ Where these treatments fail, there are several surgical techniques available. There is no current consensus on the superiority of any technique over others based on the last Cochrane systematic review in 2015.^6^ In a previous systematic review and network meta-analysis, we recommended simple trapeziectomy without any added soft tissue procedures based on our results, with some other interventions such as arthrodesis and total joint arthroplasty (TJA) showing promising results, without, however sufficient evidence.^7^ With the increasing use of TJA for TMCJ arthritis, some high-quality evidence in the form of randomized controleld trials (RCTs) has recently emerged; therefore, we decided to update our systematic review with these new data.^8^, ^9^, ^10^

The aim of the present systematic review and network meta-analysis was to identify, present, and analyze the highest-quality evidence assessing the comparative efficacy of surgical interventions for TMCJ osteoarthritis to guide both surgeons and patients.

## Materials and Methods

The present systematic review and network meta-analysis was conducted and authored according to the Preferred Reporting Items for Systematic Reviews and Meta-Analyses-Network Meta-Analysis (NMA) guidelines.^11^ A protocol was written and registered on the PROSPERO website (CRD42023467937). Our patients-interventions-comparators-outcomes were defined as follows: (1) patients with TMCJ arthritis (any stage/severity); (2) any type of surgical intervention; (3) any other type of surgical intervention; and (4) pain (primary outcome); functional disability, key pinch strength, and complications (secondary outcomes).

Follow-up was defined as: (1) short-term (≤2 years), (2) mid-term (2–5 years), and (3) long-term (>5 years). A longer than usual short-term follow-up period was chosen for this study as, in the context of prostheses, mid- and long-term data that are predominantly linked to survivorship and complications are mostly relevant years after implantation.

### Eligibility

Studies were included if they had a parallel randomized design (blinded and nonblinded) and compared any surgical procedure for TMCJ arthritis with any other surgical procedure. No criteria were applied for the severity of arthritis, length of the follow-up, and postoperative rehabilitation protocol; however, these parameters were taken into account when pooling results based on clinical homogeneity. Non-English, nonhuman, nonrandomized studies and studies with participants less than 18 years of age were excluded.

### Search strategy—screening

A thorough literature search was conducted via Medline, EMBASE, Scopus, and the Cochrane Database from inception to October 2023. The following search terms were used in “all fields”: “((((thumb) OR (carpometacarpal)) OR (trapeziometacarpal)) OR (rhizarthrosis) OR (basilar)) AND (arthritis)) AND (surgery).”

Relevant review articles were screened to identify eligible articles that may have been missed in the initial search. Additionally, reference list screening and citation tracking in Google Scholar were performed for each eligible article. The gray literature was searched via Open Gray for unpublished studies to minimize the risk of publication bias.

Figure S1, available online on the Journal’s website at https://www.jhsgo.org (Preferred Reporting Items for Systematic Reviews flowchart) illustrates the article screening process.

### Risk of bias assessment—grading of certainty of evidence

Internal validity (freedom from bias) was assessed separately by two authors (D.C. and A.H.) and a third opinion (N.N.) was sought where disagreements existed. The “Cochrane Collaboration’s tool for assessing risk of bias in randomized trials” was used.^12^ Overall risk of bias for each study was determined by the authors with the use of judgment regarding the likelihood of the present biases influencing the true results of the study. Justifications are presented for all decisions. The certainty (strength) of the evidence was graded by the GRADE-NMA tool.^13^ The certainty of the evidence was graded separately for each outcome measure.

### Data extraction

The results for outcomes that were quantitatively pooled were considered significant if they were both statistically and clinically significant. As clinical significance, we defined a difference of at least 1.0 points in VAS for pain (0–10 scale), 11 points in disabilities of the arm, shoulder, and hand (DASH), and 0.9 kg for key pinch strength (no relevant literature, set using the half pooled standard deviation of contributing data).” Complications were categorized as minor (eg, sensory nerve paresthesia, tendon-related pathology, scar tenderness, etc) or major (eg, any pathology requiring revision surgery, persistent CRPS, etc).^14^, ^15^, ^16^

Finally, a NMA was conducted for short-term follow-up ranking surgical interventions according to their likelihood of being the most effective for each outcome measure. Network meta-analysis is a statistical technique for comparing three or more interventions simultaneously in a single analysis by combining both direct and indirect evidence across a network of studies. It produces estimates of the relative effects between any pair of interventions in the network, and it allows estimation of the ranking of interventions.^17^ Surface under the cumulative ranking curve (SUCRA) diagrams were produced to demonstrate the probability of each included treatment being the most effective, and league tables showing the results of the comparison of each included treatment to all other treatments were also created, with MDs/SMDs and 95% confidence intervals. The certainty of evidence for the ranks was assessed using the GRADE-NMA tool.^13^

### Statistical analysis

The Review Manager V.5 (RevMan) software application was used for pairwise meta-analyses and their accompanying forest plots, *P* values, and heterogeneity tests (χ2 and I^2^). Mean differences (MD) with 95% confidence intervals (CI) were calculated and reported for continuous outcome measures where outcome measures were identical in the studies, whereas standardized mean differences (SMD) were used where these were similar but not identical. Odds ratios (OR) with 95% CI were calculated for dichotomous outcomes (complications). Expecting wide variability in studies’ settings, random-effects models were deployed for meta-syntheses.

STATA 16.1 with Ian White’s “mvmeta” extension (multivariate random-effects meta-regression) was used for network meta-analyses (frequentist approach).^18^

## Results

### Characteristics of included studies

A total of 21 RCTs were eligible and were included in the present review. Table 1 summarizes their characteristics. The 21 eligible studies had a total of 1,726 participants (mean age: 57.6 years). 8, 9, 10^,^^19^, ^20^, ^21^, ^22^, ^23^, ^24^, ^25^, ^26^, ^27^, ^28^, ^29^, ^30^, ^31^, ^32^, ^33^, ^34^, ^35^, ^36^ The following types of surgical interventions participated in pairwise and network meta-analyses: simple trapeziectomy (n = 9), Silicone (Swanson) arthroplasty (n = 1), trapeziectomy with autograft tendon interposition (TI) with or without ligament reconstruction (LR; n = 13 LRTI, n = 2 TI only), trapeziectomy with LRTI using allograft (n = 1), Artelon joint spacer (n = 1), trapeziometacarpal arthrodesis (n = 1), and TJA (n = 5; cementless n = 4, cemented n = 1; single-mobility n = 3, dual-mobility n = 2). Swanson arthroplasty and Artelon spacer were treated as separate interventions to TJA, and their results were considered separately. Where a tendon autograft was used for TI, the following tendons were used: flexor carpi radialis (n = 16), abductor pollicis longus (n = 4), palmaris longus (n = 2), and extensor carpi radialis brevis (n = 1). Final follow-up ranged from 12 months to 18 years. Publication dates ranged from 1997 to 2023.

**Table 1:** Characteristics of Included Randomized Studies

Authors (Date)	Population Size, Mean Age (Range), %F	Interventions Assessed	Outcome Measures	Follow-Up
Davis et al^35^ (2004)	n = 183, 59 y (40–82), 100% F	1 Trapeziectomy n = 62 2 Trapeziectomy with LRTI n = 62 3 trapeziectomy with palmaris longus interposition n = 59	1 Pain (with use, rest with restriction, rest no restriction, rest some restriction, and night pain) 2 Strength (grip, key pinch, and tip pinch) 3 ROM 4 Complications	3 mo, 1 y
Davis and Pace^36^ (2009)	n = 128, 60 y (44–81), 84.37% F	1 Trapeziectomy n = 67 2 Trapeziectomy with LRTI n = 61	1 Pain (DASH, PEM) 2 Strength (grip, key pinch, and tip pinch) 3 ROM 4 Complications	3 mo, 1 y
Davis et al^33^ (1997)	n = 76, 58 y (range not available), 100% F	1 Trapeziectomy n = 30 2 Trapeziectomy with TI (palmaris longus) n = 23 3 Trapeziectomy with LRTI (palmaris longus) n = 23	1 Pain 2 Functional disability 3 Strength (grip, key pinch, and tip pinch) 4 ROM 5 Joint imaging (trapeziometacarpal distance) 6 Complications	3, 12 mo
Gerwin et al^19^ (1997)	n = 20, 62 y (range not available), %F not available	1 Trapeziectomy with LRTI (FCR) n = 9 2 Trapeziectomy with LR n = 11	1 Function (VAS 0–100) 2 ROM 3 Strength (lateral pinch, 2-point pinch, 3-point chuck pinch, and grip) 4 Joint imaging (trapeziometacarpal distance)	Mean 23 mo
Belcher and Nicholl^20^ (2000)	n = 42, 61 y (range not available), 88% F	1 Trapeziectomy with LRTI (APL) n = 23 2 Trapeziectomy n = 19	1 Pain (VAS 0–10) 2 Function (VAS 0–10) 3 ROM 4 Strength (grip, pulp and key pinch) 5 Joint imaging (scapho-metacarpal and trapeziometacarpal distance) 6 Adverse events	Median 13 mo (7–29)
Tagil and Kopylov^21^ (2002)	n = 28, 62 y (48–75), 92% F	1 Trapeziectomy with TI (APL) n = 14 2 Trapeziectomy with Swanson silicon trapezium implant n = 14	1 Pain (VAS 0–100) 2 ROM 3 Strength (grip, thumb tip, key pinch) 4 Joint imaging (scapho-metacarpal distance) 5 Adverse events	Group 1: Mean 41 mo (23–66) Group 2: Mean 45 mo (22–66)
Kriegs-Au et al^22^ (2004)	n = 31, 59 y (42–75), 81% F	1 Trapeziectomy with LRTI (FCR) n = 16 2 Trapeziectomy with LR n = 15	1 Pain (never, occasional, frequent, constant) 2 Function (activities of daily living questionnaire; 1–5) 3 Buck-Gramcko score (0–56) 4 ROM 5 Strength (tip pinch, grip) 6 Joint imaging (scapho-metacarpal distance and arthroplasty index)	Mean 48.2 mo (32–64)
Hart et al^23^ (2006)	n = 40, 59 y (49–75) 65% F	1 Trapeziectomy with LRTI (FCR) n = 20 2 Arthrodesis n = 20	1 Buck-Gramcko score (0–56)	Mean 6.8 y (2–10)
Field and Buchanan^24^ (2007)	n = 65, 55 y (49–75) 86% F	1 Trapeziectomy n = 32 2 Trapeziectomy with LRTI (half FCR) n = 33	1 ROM 2 Strength (grip, key pinch, tip pinch) 3 Pain (VAS 0–10) 4 Joint imaging (scapho-metacarpal distance, height ratio, height loss) 5 Willingness to have procedure again 6 Adverse events	12 mo
Nilsson et al^25^ (2010)	n = 109, 60 y (42–83) 85% F	1 Artelon CMC Spacer n = 72 2 Trapeziectomy with TI (APL, ECRL or FCR) n = 37	1 Pain (VAS 0–10) 2 Strength (tripod pinch, key pinch, grip) 3 ROM 4 Function (DASH 0–100) 5 Satisfaction (1–5) 6 Joint imaging (trapeziometacarpal distance, subluxation of the first metacarpal, degree of osteoarthritis in the thumb CMC joint) 7 Adverse events	12 mo
Gangopadhyay et al^34^ (2012)	n = 153, 57 y (44–75), 100% F	1 Trapeziectomy n = 53 2 Trapeziectomy with TI (Palmaris Longus) n = 46 3 Trapeziectomy with LRTI (FCR) n = 54	1 Pain (0–6) 2 Restriction of activity 3 Strength (grip, key pinch, and tip pinch) 4 ROM 5 Adverse events	Median 6 y (5–18)
Salem and Davis^26^ (2012)	n = 114, 61 y (44–81) 85% F	1 Trapeziectomy with LRTI (FCR) n = 59 2 Trapeziectomy n = 55	1 Pain 2 Strength (key pinch, tip pinch, and grip) 3 Stiffness 4 ROM 5 Function (DASH 0–100) 6 Satisfaction (patient evaluating measure questionnaire)	1 y
Hansen and Stilling^27^ 2013	n = 28, 58 y (40–77) 82% F	1 Cemented joint replacement (DLC all -poly) n = 14 2 Cementless joint replacement (Elektra) n = 14	1 Pain (VAS 0–10) 2 Function (DASH 0–100) 3 Strength (grip) 4 Stereoradiographs	3, 6, 1, 24mo
Vermeulen et al^28^ (2014)	n = 38, 59 y (range not available), 100% F	1 Trapeziectomy with LRTI (FCR) n = 21 2 Arthrodesis n = 17	1 Pain (PRWHE 0–100) 2 Function (PRWHE 0–100, DASH 0–100) 3 ROM 4 Satisfaction 5 Strength (key pinch, tip pinch, three-point pinch, and grip) 6 Complications	3 mo, 1 y
Vermeulen et al^28^ (2014)	n = 72, 64 y (range not available), 100% F	1 Trapeziectomy with LRTI (FCR) – bone tunnel n = 36 2 Trapeziectomy with LRTI (FCR) – no bone tunnel n = 36	1 Pain (PRWHE 0–100) 2 Function (PRWHE 0–100, DASH 0–100) 3 Satisfaction 4 Joint imaging (trapeziometacarpal distance) return to work 5 ROM 6 Complications	3 mo, 1 y
Corain et al^30^ (2016)	n = 120, 63 y (45–77), 78% F	1 Trapeziectomy with TI (APL) n = 64 2 Trapeziectomy with K-wire stabilization n = 56	1 ROM (Kapandji test) 2 Strength (grip, key pinch, tip pinch, tripod pinch) 3 Pain (VAS 0–10) 4 Function (DASH 0–100) 5 Joint imaging (trapeziometacarpal distance) 6 FCR tendinopathy	Mean 6.8 y
Marks et al^31^ (2017)	n = 60, 65 y (range not available), 85% F	1 Trapeziectomy with LRTI (FCR) n = 29 2 Trapeziectomy with LRTI (allograft) n = 31	1 Function (MHQ 0–100, DASH 0–100) 2 Pain (MHQ 0–100) 3 QoL (SF-12) 4 Strength (key pinch, grip) 5 ROM (Kapandji score) 6 Joint imaging (trapeziometacarpal distance) 7 Complications	6 wk, 3 mo, 6 mo, 12 mo
Thorkildsen and Rokkum^32^ (2019)	n = 40, 63 y (36–77), 70% F	1 Trapeziectomy with LRTI (FCR) n = 20 2 TCMC prosthesis (Elektra) n = 20	1 Function (qDASH 0–100) 2 Pain (Nelson score 0–100) 3 ROM (Kapandji score) 4 Strength (grip, key pinch, and tip pinch) 5 Complications 6 Radiological assessment	3, 6, 12, 24 mo
Klim et al^9^ (2023)	n = 168, 58 y (53–68), 80.95% F	1 TCMC prosthesis (Stryker Moovis) n = 82 2 Trapeziectomy with LRTI (FCR) n = 86	1 Function (DASH 0–100) 2 Pain (VAS 0–10) 3 ROM (Kapandji score) 4 the hospital anxiety and depression scale (HADS) 5 Pain (SFMcGill score)	1 d,2 wk, 6 wk, 3 mo, 6 mo, 12 mo
Guzzini et al^8^ (2023)	n = 149, 67.5 y, 80% F	1 Trapeziectomy with LRTI n = 75 hands (71 patients) 2 TCMC prosthesis (Kerimedical Touch) n = 75 hands (68 patients)	1 Pain VAS 2 DASH 3 Kapandji test 4 ROM 5 Strength 6 Tip and key pinch 7 Radiological assessment	1, 3, 6, 12, 24mo
de Jong et al^10^ (2023)	n = 62, 60 y, ?%F	1 Trapeziectomy n = 31 2 TCMC prosthesis (Maia) n = 31	1 MHOQ 2 DASH 3 ROM 4 Kapandji score 5 Strength (grip, key pinch, pinch, and three-point pinch) 6 Return to work 7 Satisfaction	3, 12 mo

APL, abductor pollicis longus; CMC, carpometacarpal; FCR, flexor carpi radialis; LR, ligament reconstruction; LRTI, ligament reconstruction and tendon interposition; MHQ, Michigan hand outcomes questionnaire; PRWHE, patient-rated wrist and hand evaluation; ROM; SF-12, short form 12; TCMC, total carpometacarpal; TI, tendon interposition; Qol, quality of life; VAS, visual analog scale.

### Risk of bias—certainty of evidence

All but one study were classified as “high” overall risk of bias because of nonblinded patients. As a result, the certainty of evidence could be “moderate” at best, unless there was a reason for it to be upgraded (eg, large magnitude of effect).^10^ Risk of bias assessment results for each study can be seen in Table S1 and certainty of evidence assessment results for each pairwise meta-analysis in Table S2, available online on the Journal’s website at https://www.jhsgo.org.

### Direct comparisons of interventions (pairwise meta-analyses)

#### Trapeziectomy versus trapeziectomy with LRTI

**Figure 1:**
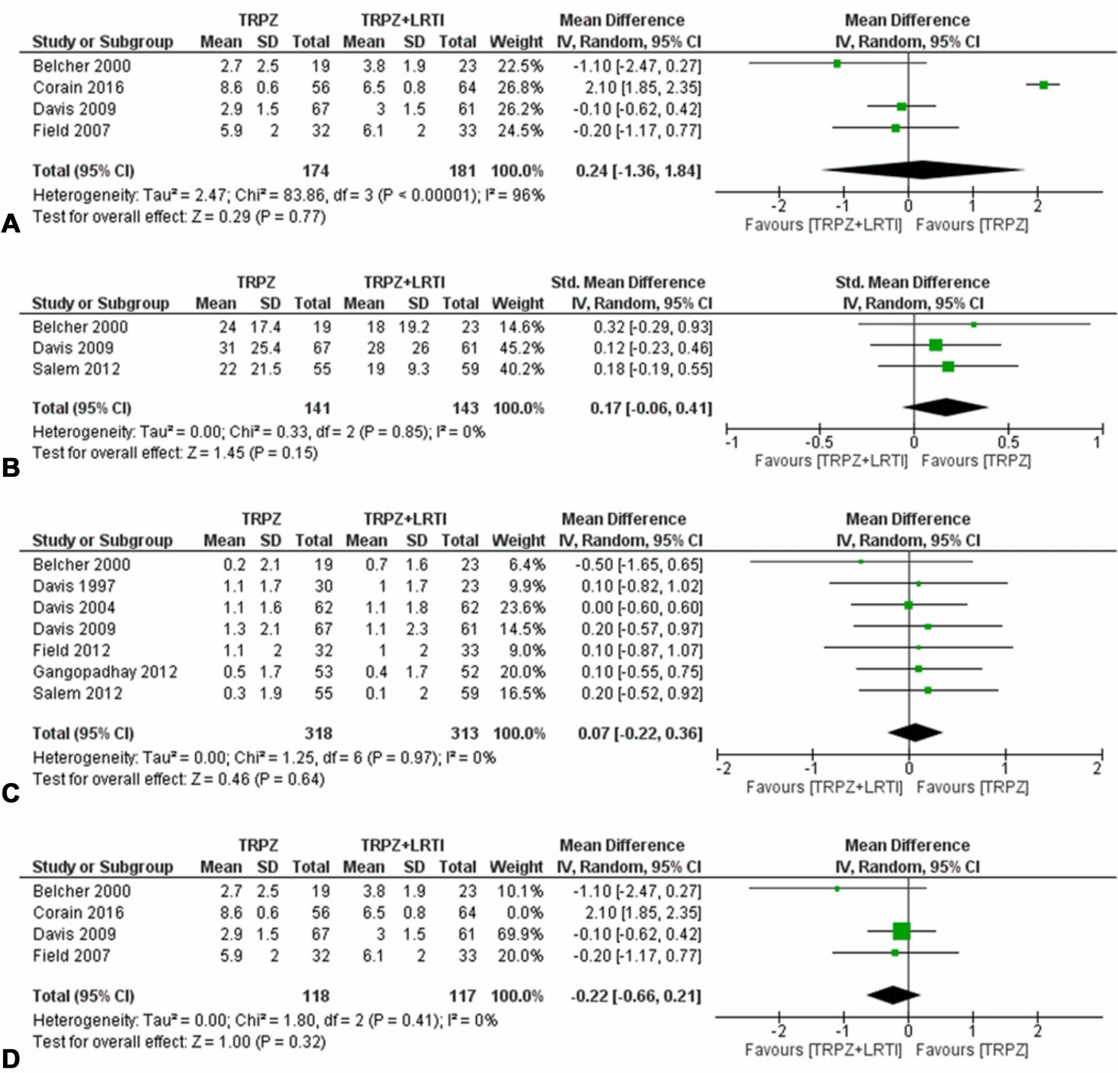
(**A-D**) Pairwise meta-analyses of the comparison between simple trapeziectomy versus trapeziectomy with LRTI for short-term pain (**A**), functional disability (**B**), key pinch strength (**C**), and pain with study responsible for statistical heterogeneity removed (**D**). TRPZ, simple trapeziectomy.

Seven studies of “high” overall risk of bias assessed short-term outcomes of trapeziectomy alone compared with trapeziectomy with LRTI (Fig. 1A–D).^20^^,^^24^^,^^26^^,^^33^, ^34^, ^35^, ^36^ Pairwise meta-analyses for pain, functional disability, and key pinch strength demonstrated no statistically or clinically significant benefit of one intervention over the other [pain VAS, MD 0.24 points (−1.36 to 1.84), *P* = .77, 4 studies, 355 patients; low certainty of evidence, Figure 1A; functional disability, SMD 0.17 (−0.06 to 0.41), *P* = .15, 3 studies, 284 patients; moderate certainty of evidence, Figure 1B; key pinch, MD 0.07 kg (−0.22 to 036]), *P* = .64, 7 studies, 631 patients; moderate certainty of evidence, Fig. 1C]. For pain, sensitivity analyses identified and removed the study that was responsible for inconsistency (statistical heterogeneity, I^2^ = 96%), which downgraded the certainty of evidence by two levels.^30^ When the pairwise meta-analysis was repeated without it, the numerical results were similar showing no difference between the two interventions; however, the certainty of evidence of this result was now moderate [MD 0.22 points (−0.66 to 0.21]), *P* = .32, 3 studies, 236 patients, Fig. 1D].

#### Trapeziectomy (with or without LRTI) versus TJA

**Figure 2:**
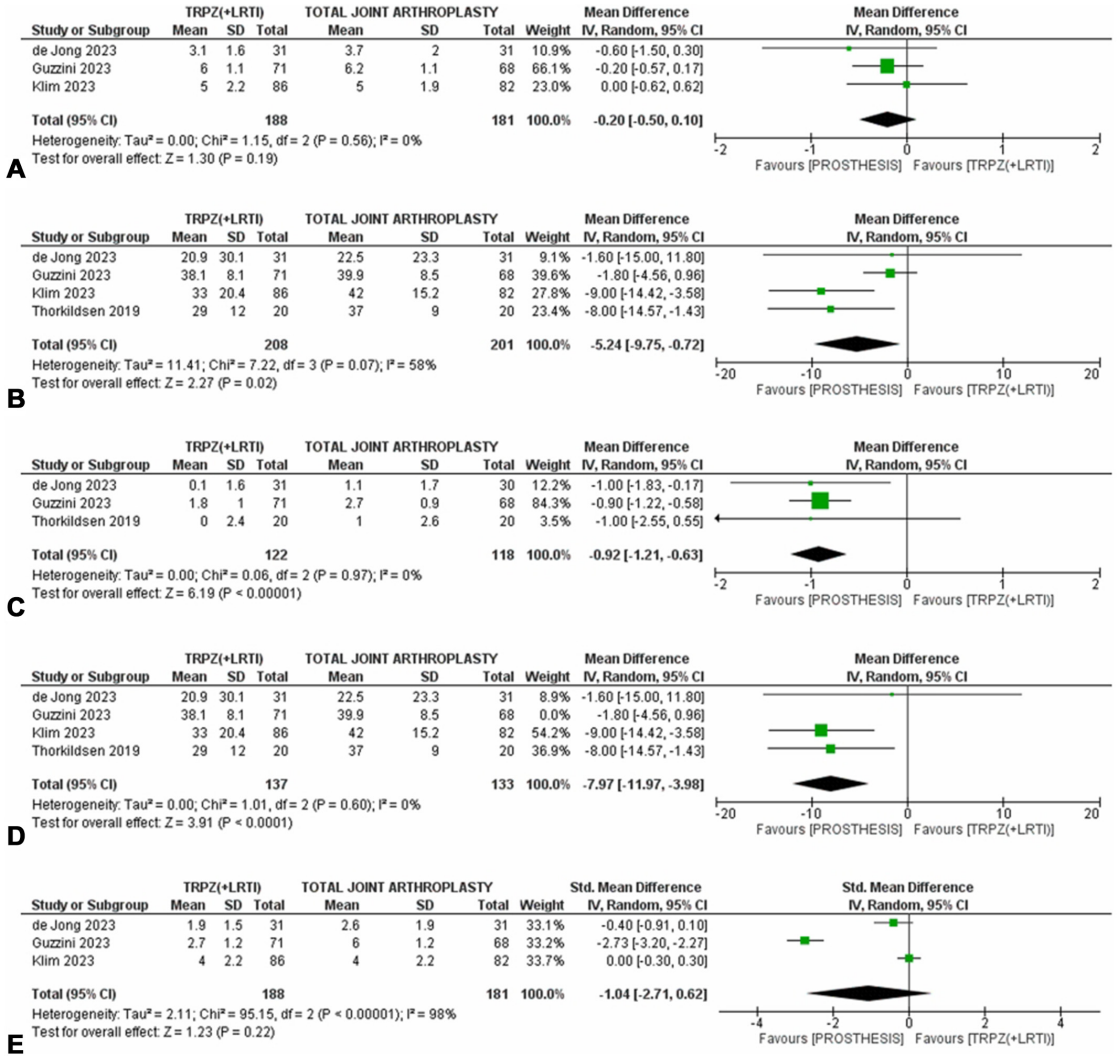
(**A-E**) Pairwise meta-analyses of the comparison between trapeziectomy with or without LRTI and TJA for short-term pain (**A**), functional disability (**B**), key pinch strength (**C**), pain with study responsible for statistical heterogeneity removed (**D**), and short-term pain (**E**). TRPZ, simple trapeziectomy.

Four studies compared TJA with simple trapeziectomy only (n = 1)^10^ or trapeziectomy with LRTI (n = 3; Fig. 2A–D). One was of “low” and three “high” overall risk of bias.^8^, ^9^, ^10^^,^^32^ Given the similar outcomes of simple trapeziectomy versus trapeziectomy with LRTI reported above, these were grouped together for their comparison with TJA to increase the amount of pooled data. Short-term pain was similar between the two interventions [pain VAS, MD 0.20 points (−0.10 to 0.50), *P* = .19, 3 studies, 369 patients; moderate certainty of evidence, Fig. 2A). Short-term functional disability (DASH) and key pinch strength had greater improvements with TJA compared with trapeziectomy that reached statistical significance [DASH, MD 5.24 points (0.72–9.75), *P* = .02, 4 studies, 409 patients, low certainty of evidence, Figure 2B; key pinch strength, MD 0.92 kg (0.63–1.21), *P* < .001, 3 studies, 240 patients, moderate certainty of evidence, Fig. 2C]. Only the difference in key pinch strength exceeded clinical significance. For functional disability, we performed sensitivity analyses to identify the study that was responsible for high statistical heterogeneity (I^2^ = 58%). When the meta-analysis was repeated without the data from this study, the numerical difference between the two interventions was slightly greater, still statistically but not clinically significant, but this time the certainty of evidence was moderate as it was not downgraded for inconsistency (statistical heterogeneity) [DASH, MD 8.0 points (4.0–11.0), *P* < .01, 3 studies, 270 patients, Fig. 2D].^8^

For the primary outcome measure (pain), we also performed a pairwise meta-analysis for early short-term follow-up (12 weeks), and, based on evidence of low certainty, there was a clinically significant difference favouring TJA over trapeziectomy, which was however statistically non-significant [pain, SMD 1.04 (−0.62 to 2.71), *P* = .22, 3 studies, 369 patients; Fig. 2E].

No other quantitative analyses were possible for mid- or long-term outcomes.

Table S2 summarizes the GRADE assessment results for the pairwise meta-analyses.

### Complications

A total of 17 studies reported complications of their assessed surgical interventions. The complications reported in the included studies are listed in Table S3, available online on the Journal’s website at https://www.jhsgo.org. Pairwise meta-analyses were possible for the following comparisons:

#### Trapeziectomy versus trapeziectomy with LRTI

**Figure 3:**
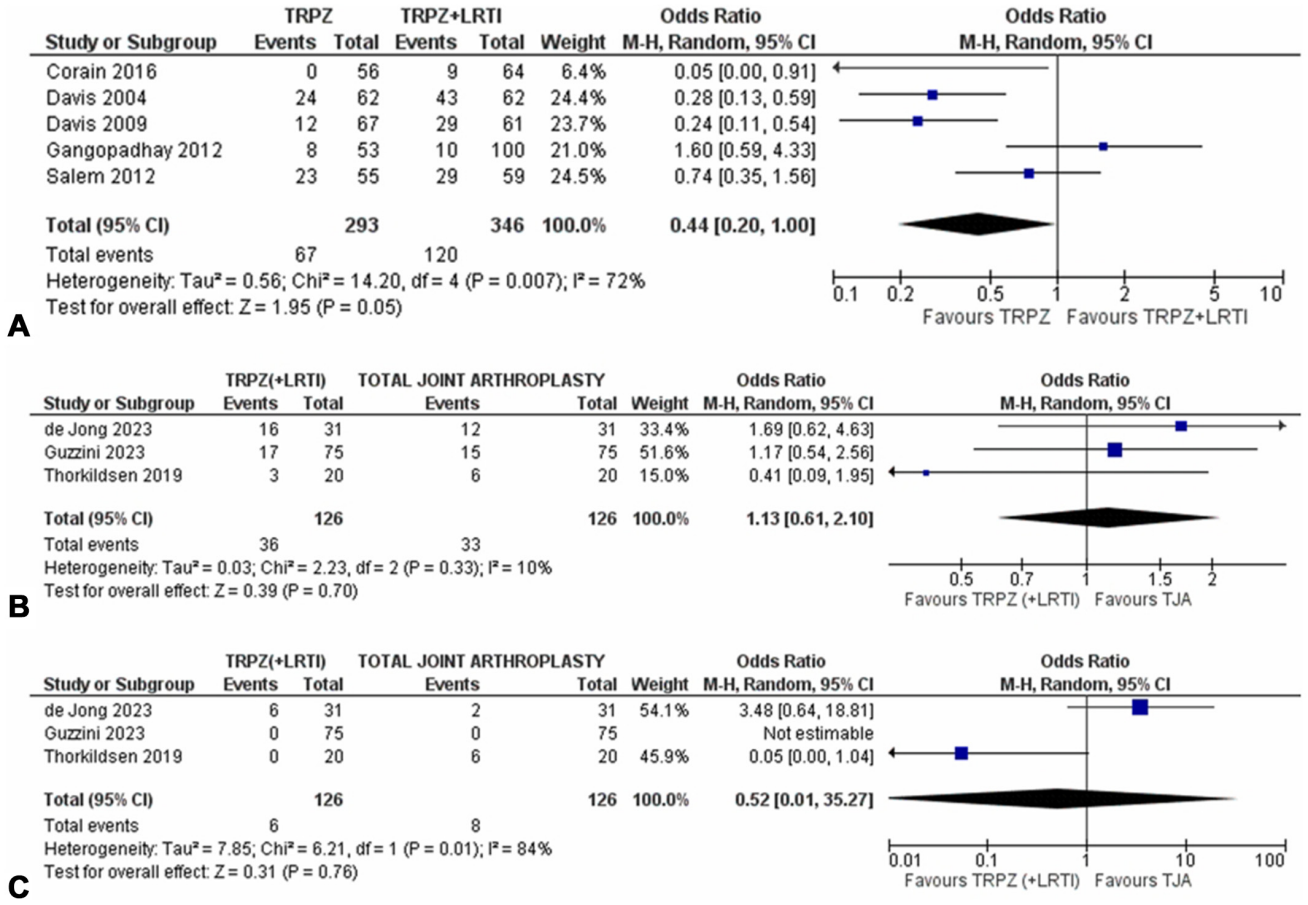
(**A-C**) Pairwise meta-analyses for short-term complications for the comparison between simple trapeziectomy versus trapeziectomy with LRTI for total complications (**A**) and trapeziectomy (± LRTI) versus TJA for total complications (**B**) and major complications (**C**). TRPZ, simple trapeziectomy.

For total complications, the meta-analysis showed no differences between the two interventions [OR 0.44, CI (0.2–1.0), *P* = .05, 5 studies, 187 patients, low certainty of evidence; Fig. 3A]. All complications were minor except for five cases of CRPS which persisted for 1 year (n = 3 in simple trapeziectomy, n = 2 in trapeziectomy with LRTI).

#### Trapeziectomy (± LRTI) versus TJA

For both total complications and major complications, the two interventions were found to have no significant differences [OR 1.13, (0.61–2.10), *P* = .70, 3 studies, 252 patients; OR 0.52, (0.01–35.27), *P* = .76, 3 studies, 252 patients, respectively, low certainty of evidence for both; Fig. 3B, C). Major complications included n = 6 reoperations for symptomatic metacarpal collapse in the trapeziectomy group (n = 6) and n = 3 dislocations, n = 2 cup loosening, n = 1 deep infection, n = 1 revision (reason not specified), and n = 1 subsequent thumb metacarpophalangeal joint arthrodesis in the TJA group. The RCT by Klim et al was not included in the meta-analyses as complications in the trapeziectomy group were not reported; in the TJA group, they reported three revisions (n = 2 for dislocation, n = 1 cup loosening).

### Network meta-analyses (direct and indirect comparisons of interventions)

**Figure 4:**
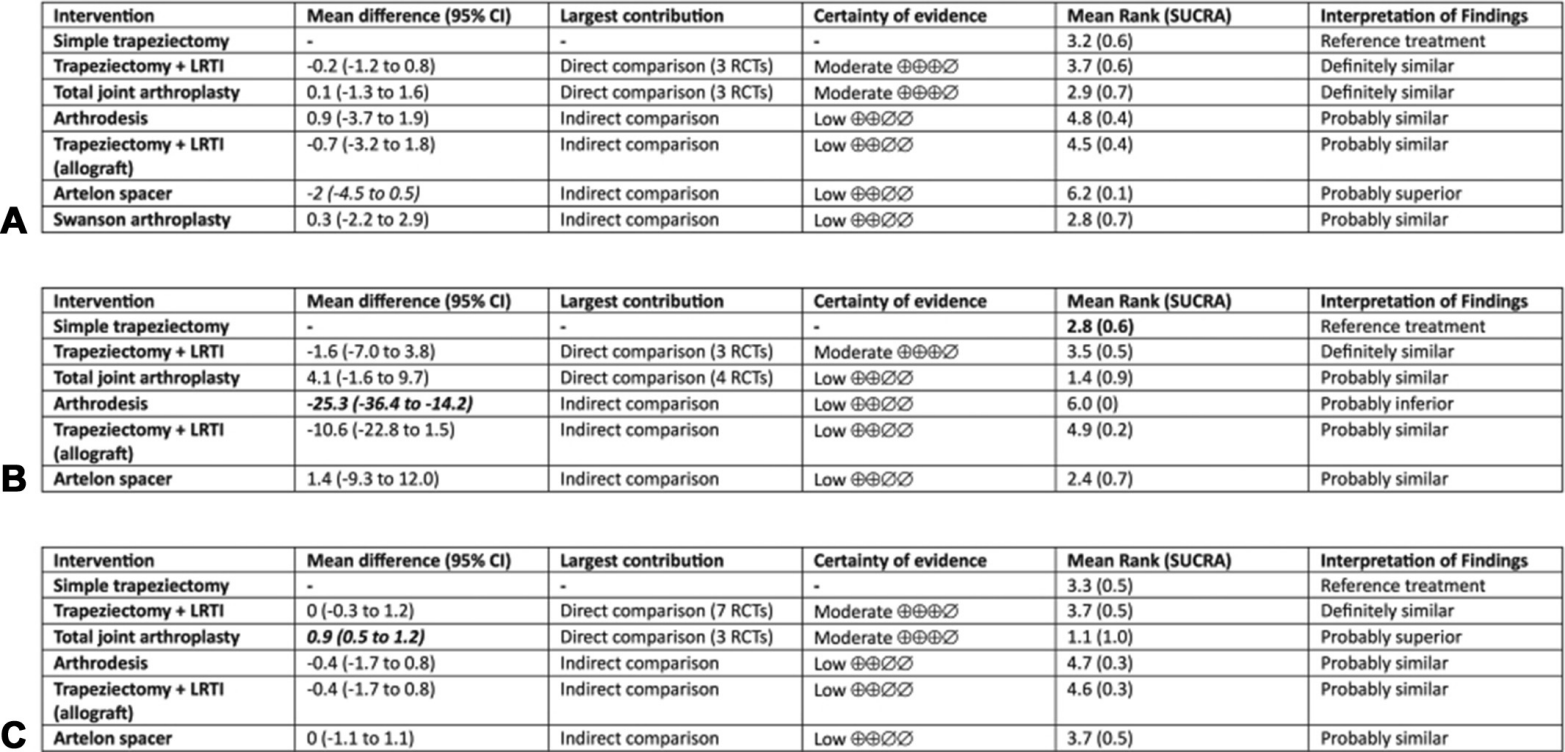
Summary of findings table showing the result of the comparison of each participating interventions with the reference intervention (simple trapeziectomy) for short-term pain (**A**), functional disability (DASH scale, **B**), and key pinch strength (**C**) deriving from the NMA. The interpretation of findings decision was based on significance of the mean difference (statistical and clinical) and the certainty of evidence.

A total of 13, 12, and 14 studies were used in network meta-analyses for short-term pain, function, and key pinch strength, respectively. The network maps, rank bars, SUCRA, and league tables for pain, function, and key pinch strength are discussed (Tables S4–S6 and Figs. S2–S4, available online on the Journal’s website at https://www.jhsgo.org). The summary of findings tables for short-term patient-reported pain, function, and key pinch strength summarizing the estimates of the comparisons of each participating intervention with the reference intervention (simple trapeziectomy) are shown in Figure 4A–C.

### Probability ranks and league tables

- *Short-term pain:* Swanson arthroplasty and TJA had the highest probability of being the most effective surgical treatments for pain relief (SUCRA 0.7 for both).
In the league table, no comparisons produced differences at statistical significance; however, the following comparisons showed differences at clinical significance (all favoring the intervention stated first): TJA versus arthrodesis, Swanson arthroplasty versus arthrodesis, and all interventions versus Artelon spacer.
- *Short-term functional disability:* TJA had the highest probability of being the most effective treatment modality for functional disability (SUCRA 0.9).
In the league table, all interventions were superior to arthrodesis at statistical and clinical significance. TJA was superior to trapeziectomy with LRTI (both autograft tendon and allograft) statistically, but only its comparison with allograft LRTI reached clinical significance.
- *Short-term key pinch strength:* TJA had the highest probability of being the most effective surgical treatment for key pinch strength (SUCRA 1.0).
In the league table, TJA was superior to all other interventions (except Artelon spacer, no difference) at both statistical and clinical significance. No other significant differences were observed.

## Discussion

In this systematic review and NMA on surgical treatment of TMCJ osteoarthritis, we demonstrated once again the lack of superiority of adding a TI procedure to simple trapeziectomy, which has been previously reported.^7^ Second, based on mostly newly published data, we found that TJA may be superior to trapeziectomy for short-term key pinch strength. Short-term pain relief and functional disability improvements between the two were observed to be similar; however, pain relief at up to 3 months after surgery may be greater with TJA. Finally, among all included surgical interventions, TJA had the highest probability of ranking first for all short-term pain, functional disability, and key pinch strength in the network meta-analyses, with evidence of low/very low certainty, and short-term complication profiles between simple trapeziectomy and trapeziectomy with LRTI, and trapeziectomy and TJA were not significantly different.

Treatment of TMCJ arthritis with TJA is not new. Historically, the use of prostheses was linked to frequent major complications, including dislocations and cup loosening; however, these appear to be less of an issue with newer prostheses predominantly, thanks to their dual-mobility nature.^37^^,^^38^ This is evident in the systematic review by Holme et al^39^ which showed that the Moovis (Stryker) implant, the only dual-mobility prosthesis included in the review, had the lowest dislocation and cup loosening rates (0.4% for both at mean 48 months follow-up). Loosening rates in other (single-mobility) implants were as high as 38.2% (de la Caffinière, implant, mean follow-up 68 months) and dislocation rates as high as 6.8% (ARPE implant, mean follow-up 78 months). In the RCT by Guzzini et al^8^ included in the present review, there were zero reported cup loosenings or dislocations at 2 years follow-up with the dual-mobility Touch prosthesis (KeriMedical); however, this may not accurately reflect the complication profile and reoperation rates of the implant at longer follow-up.

A recently published systematic review predominantly including retrospective studies estimated the failure rates of various arthroplasty and nonarthroplasty implants.^40^ The authors reported significantly lower failure rates in the nonimplant arthroplasty groups (trapeziectomy with or without ligament reconstruction and/or TI; 0.24 failures per 100 procedure years) compared with implant arthroplasty groups (0.49 failures per 100 procedure years).^39^ Similarly, in an analysis of a national cohort of 43,076 surgeries for TMCJ arthritis, the authors reported that further surgery following a primary procedure was most likely with TJA (3.84%) and least likely with simple trapeziectomy (1.12%).^41^ However, both of these studies were published before the prevalent use of dual-mobility implants using data predominantly from single-mobility prostheses. Finally, survivorship data of single-mobility TJA (IVORY, ARPE) are promising with 10-year survivorship ranging between 85% and 93% in previously published studies; long-term survivorship data of dual-mobility implants are awaited.^42^, ^43^, ^44^

The lack of and need for high-quality, comparative long-term safety data are an important consideration. The most striking example is the Swanson silicone implant, which is not used anymore for the management of TMCJ arthritis because of previously reported high reoperation rates secondary to implant breakage and dislocation and silicone synovitis.^45^ However, in the small RCT included in our review, it performed as well as trapeziectomy with LRTI for short-term pain and strength, and it ranked first for short-term pain in our NMA, which would make it a promising intervention in the absence of long-term safety data.^21^ An additional factor that should also not be ignored is cost; surgeons, patients, and health care systems need to consider both short-term costs related to the direct cost of the implant as well as indirect long-term costs related to potential complications and reoperations when considering the use of TJA for its possible, yet uncertain (largely clinically insignificant) benefits compared with the consistent and reliable trapeziectomy.

Our study has limitations. Despite its methodological rigor with a detailed systematic search, thorough risk of bias, and certainty of evidence assessments, the main limitation is secondary to the included RCTs themselves, which were deemed to be of high overall risk of bias because of the failure to blind their participants. Even in those RCTs that claimed to have used blinded assessors, the assessment of outcome was technically not blinded as the real assessors of patient-reported outcome measures (pain and functional disability) were the patients themselves, who were unblinded. Additionally, some of the surgical treatments were only represented by single (high risk of bias) RCTs in the NMA, which downgraded the certainty of the evidence of the results, and some other not uncommonly used surgical interventions, such as denervation, suture suspensionplasty, and arthroscopic techniques, were not included at all as they have not been studied in RCTs yet. Finally, there was some heterogeneity across studies in terms of techniques used and follow-up time points, which has been accounted for in the certainty of evidence assessment.

We recommend that simple trapeziectomy remains the gold standard surgical treatment for TMCJ arthritis. When TJA is offered, the surgeon should counsel the patient appropriately and without bias, explaining both the possible, yet uncertain, benefits of prostheses, which relate to earlier pain relief and superior short-term strength, but also the higher rates of possible complications, which may necessitate further surgery and the lack of high-quality long-term data.

## Conflicts of Interest

No benefits in any form have been received or will be received related directly to this article.
