# Supplementary material for: Surgery for Trapeziometacarpal Joint Arthritis: A Network Meta-Analysis of Randomized Studies
Source: J Hand Surg Glob Online. 2025 May 22;7(4):100737. doi: 10.1016/j.jhsg.2025.100737 (PMC12150098; doi:10.1016/j.jhsg.2025.100737)
Supplement: Supplementary Tables 1-6 and Supplementary Figure 1-4 [file mmc1.docx]

**Supplementary Material**

**Tables**

| First Author (year) | Internal Validity  (Cochrane’s Collaboration Tool for Assessing Risk of Bias) | | | | | | | Overall risk |
| --- | --- | --- | --- | --- | --- | --- | --- | --- |
|  | *Selection*  *bias* | | *Performance*  *bias* | *Detection*  *bias* | *Attrition*  *bias* | *Reporting*  *bias* | *Other* |  |
|  | *Random sequence generation* | *Allocation concealment* | *Blinding of patients and staff* | *Blinding of outcome measures* | *Completeness of outcome data* | *Selective reporting* |  |  |
| Davis (1997) | High (stratified) | High (not blinded) | High (not blinded) | High (not blinded) | Low | High (not all results reported) | High (no sample size calculation, no p values, additional procedures) | **High** |
| Gerwin (1997) | ? | ? | High (not blinded) | High (not blinded) | ? | ? | ? | **High** |
| Belcher (2000) | ? | ? | High (not blinded) | High (not blinded) | ? | Low | High (no sample size calculation) | **High** |
| Tagil (2002) | ? | ? | High (not blinded) | High (not blinded) | Low | Low | Low | **High** |
| Davis (2004) | Low | Low | High (not blinded) | High (not blinded) | Low | Low | Low | **High** |
| Kriegs-Au (2004) | Low | Low | High (not blinded) | High (not blinded) | Low | High (no variability statistics) | High (no sample size calculation) | **High** |
| Hart (2006) | ? | ? | High (not blinded) | High (not blinded) | ? | Low | ? | **High** |
| Field (2007) | Low | ? | High (not blinded) | High (not blinded) | Low | Low | High (no sample size calculation, or baseline comparison) | **High** |
| Davis (2009) | Low | Low | High (not blinded) | High (not blinded) | Low | Low | Low | **High** |
| Nilsson (2010) | ? | ? | High (not blinded) | Low | Low | Low | High (no baseline comparison) | **High** |
| Gangopadhyay (2012) | Low | Low | High (not blinded) | High (not blinded) | Low | Low | High (only women – results may not be generalisable to men) | **High** |
| Salem (2012) | ? | ? | High (not blinded) | High (not blinded) | Low | Low | High (no baseline comparison) | **High** |
| Hansen (2013) | Low | Low | Low (patients)  High (Staff) | Low | Low | High (no comparison for most outcome measures) | High (no baseline comparison, grip strength different in groups) | **High** |
| Vermeulen (2014) | Low | ? | High (not blinded) | High (not blinded) | Low | High (no between-group statistical comparisons) | High (study prematurely terminated, sample size not enough for power calculation) | **High** |
| Vermeulen (2014a) | Low | ? | High (not blinded) | High (not blinded) | Low | Low | High (group sample sizes not enough for power calculation; only women included – results may not be generalisable to men) | **High** |
| Corain (2016) | Low | ? | High (not blinded) | High (not blinded) | ? | Low | High (no sample size calculation, | **High** |
| Marks (2017) | Low | Low | High (not blinded) | High (not blinded) | Low | Low | Low | **High** |
| Thorkildsen (2019) | Low | Low | High (not blinded) | High (not blinded) | Low | Low | High (no baseline comparisons) | **High** |
| Klim (2023) | Low | Low | High (not blinded) | High (not blinded) | Low | Low | Low | **High** |
| Guzzini (2023) | Low | ? | High (not blinded) | High (not blinded) | Low | Low | Low | **High** |
| De Jong (2023) | Low | Low | Low | Low | Low | Low | Low | **Low** |

**Suppl. Table 1**

| **Comparison** | **Outcome** | **Number of RCTs** | **GRADE parameters** | | | | | **Certainty of Evidence** |
| --- | --- | --- | --- | --- | --- | --- | --- | --- |
|  |  |  | **Overall RoB** | **Inconsistency** | **Imprecision** | **Indirectness** | **Other** |  |
| **Trapeziectomy vs Trapeziectomy with LRTI** | **Pain** | n=3 | High | Low | Low | Low | Low | Moderate ⊕⊕⊕∅ |
|  | **Functional disability** | n=3 | High | Low | Low | Low | Low | Moderate ⊕⊕⊕∅ |
|  | **Key pinch strength** | n=7 | High | Low | Low | Low | Low | Moderate ⊕⊕⊕∅ |
|  | **Total complications** | n=5 | High | Low | High | High | Low | Very Low ⊕∅∅∅ |
| **Trapeziectomy (+/- LRTI) vs total joint arthroplasty** | **Pain** | n=3 | High | Low | Low | Low | Low | Moderate ⊕⊕⊕∅ |
|  | **Functional disability** | n=4 | High | High | Low | Low | Low | Low ⊕⊕∅∅ |
|  | **Key pinch strength** | n=3 | High | Low | Low | Low | Low | Moderate ⊕⊕⊕∅ |
|  | **Total complications** | n=3 | High | Low | High | High | Low | Very Low ⊕∅∅∅ |
|  | **Major complications** | n=3 | High | Low | High | High | Low | Very Low ⊕∅∅∅ |

**Suppl. Table 2**

| Study/Procedure | Trapeziectomy | Trapeziectomy+LRTI | Total joint arthroplasty | Arthrodesis | Artelon spacer |
| --- | --- | --- | --- | --- | --- |
| Davis et al., 2004 | N=12 SRN dysfunction (N=6 persisted at 1y)  N=2 PCB median nerve dysfunction (N=2 persisted at 1y)  N=1 FCR/palmaris longus pulling (resolved)  N=5 scar tenderness (resolved)  N=3 CRPS (N=1 persisted at 1y) | N=12 SRN dysfunction (N=3 persisted at 1y)  N=5 PCB median nerve dysfunction (N=3 persisted at 1y)  N=15 FCR/palmaris longus pulling (N=8 persisted at 1y)  N=5 scar tenderness (N=3 persisted at 1y)  N=6 CRPS (N=2 persisted at 1y) |  |  |  |
| Kriegs-Au et al., 2004 | N=2 temporary paraesthesia | N=2 temporary paraesthesia  N=1 CRPS |  |  |  |
| Nilsson et al., 2005 |  |  |  |  | N=2 transient inflammatory reactions |
| Hart et al. 2006 | N=2 CRPS |  |  | N=2 CRPS  N=4 delayed union (10 weeks) |  |
| Field et al., 2007 | N=2 superficial wound infections  N=1 CRPS | N=1 superficial wound infection  N=4 CRPS  N=6 wound adherence of volar wrist wounds |  |  |  |
| Davis et al., 2009 | N=12 SRN dysfunction (N=5 persisted at 1y)  N=3 CRPS (N=2 persisted at 1y)  N=4 scar tenderness (N=2 persisted at 1y) | N=8 SRN dysfunction (N=4 persisted at 1y)  N=3 PCB median nerve dysfunction (N=1 persisted at 1y)  N=1 CRPS (resolved)  N=14 FCR pulling (N=1 persisted at 1y)  N=1 de Quervain’s  N=2 scar tenderness (N=1 persisted at 1y) |  |  |  |
| Gangopadhyay et al., 2012 | N=5 SRN dysfunction (N=2 persisted at 5y)  N=2 PCB median nerve dysfunction (N=2 persisted at 5y)  N=1 CRPS (resolved) | N=1 SRN dysfunction (N=1 persisted at 5y)  N=3 PCB median nerve dysfunction (N=3 persisted at 5y)  N=3 tendon pulling (N=2 persisted at 5y)  N=3 scar tenderness (N=2 persisted at 5y) |  |  |  |
| Salem et al., 2012 | N=12 SRN paraesthesia (N=0 persisted long-term)  N=2 FCR pulling  N=4 scar tenderness  N=4 CRPS  N=1 de Quervain’s | N=8 SRN paraesthesia (N=4 persisted long-term)  N=3 PCB median nerve parasthesia (N=3 persisted long-term)  N=14 FCR pulling  N=2 scar tenderness  N=1 CRPS  N=1 de Quervain’s |  |  |  |
| Hansen et al., 2013 |  |  | N=3 revisions for cup loosening |  |  |
| Vermeulen et al., 2014a |  | N=6 total complications  N=3 sensory disturbance  N=2 “tendinitis”  N=1 CRPS (mild) |  | N=15 total complications  N=2 non-union  N=2 CRPS (n=1 severe)  N=3 sensory disturbance  N=2 neuroma  N=3 delayed union |  |
| Vermeulen et al., 2014b |  | N=2 scar tenderness  N=1 sensory changes  N=1 infection  N=14 “tendinitis” (N=1 requiring revision surgery)  N=2 neuroma (N=1 requiring revision surgery)  N=2 mild CRPS |  |  |  |
| Corain et al., 2016 | nil | N=9 FCR tendinitis |  |  |  |
| Marks et al., 2017 |  | N=15 complications (N=5 FCR, N=10 allograft)  FCR  N=2 CRPS, N=1 trigger thumb, N=1 peristsent pain at FCR, N=1 FCR tendinitis  Allograft  N=1 CRPS, N=1 thenar atrophy and persistent pain, N=1 FCR tendinitis, N=7 partial FCR rupture |  |  |  |
| Thorkildsen et al., 2019 |  | N=3 total complications  N=1 forearm haematoma  N=1 FCR pain  N=1 persistent pain at base of thumb | N=6 total complications  N=2 cup loosenings  N=3 dislocations  N=1 deep infection |  |  |
| De Jong et al., 2023 | Perioperative N=1 FCR rupture, N=1 revision for incomplete resection  Postoperative (within 1 year)  N=16 total complications  N=6 revisions due to symptomatic metacarpal collapse  N=1 carpal tunnel syndrome, n=2 trigger thumb, n=3 adhesions |  | Perioperative N=2 converted to trapeziectomy due to trapezium fracture, one EPL rupture  Postoperative (within 1 year)  N=12 total complications  N=1 revision  N=1 Carpal tunnel syndrome, n=3 trigger finger, n=2 de Quervain’s, n=1 need for MCPJ arthrodesis |  |  |
| Guzzini et al., 2023 |  | N=7 trigger thumb  N=6 dorsal thumb paraesthesia  N=4 persistent pain | N=9 de Quervain’s  N=4 dorsal thumb paraesthesia  N=2 trigger thumb |  |  |
| Klim et al., 2023 |  | NR | N=3 revision procedures (N=2 dislocation, N=1 loosening)  N=2 de Quervain’s (required surgical release) |  |  |

**Suppl. Table 3**.

| **A - TRPZ** |  |  |  |  |  |  |
| --- | --- | --- | --- | --- | --- | --- |
| -0.2 (-1.2 to 0.8) | **B - TRPZ+LRTI** |  |  |  |  |  |
| 0.1 (-1.3 to 1.6) | 0.3 (-1 to 1.5) | **C - TJA** |  |  |  |  |
| 0.9 (-3.7 to 1.9) | -0.7 (-3.3 to 1.9) | *-1 (-3.9 to 1.9)* | **D - Arthrodesis** |  |  |  |
| -0.7 (-3.2 to 1.8) | -0.5 (-2.8 to 1.8) | -0.8 (-3.4 to 1.8) | 0.2 (-3.2 to 3.6) | **E - TRPZ+LRTI (allo)** |  |  |
| *-2 (-4.5 to 0.5)* | *-1.8 (-4.1 to 0.5)* | *-2.1 (-4.7 to 0.5)* | *-1.1 (-4.5 to 2.3)* | *-1.3 (-4.5 to 1.9)* | **F – Artelon spacer** |  |
| 0.3 (-2.2 to 2.9) | 0.5 (-1.8 to 2.8) | 0.2 (-2.5 to 2.8) | *1.2 (-2.3 to 4.7)* | 1 (-2.3 to 4.3) | *2.3 (-0.9 to 5.5)* | **G – Swanson arthroplasty** |

**Suppl. Table 4**

| **A – TRPZ** |  |  |  |  |  |
| --- | --- | --- | --- | --- | --- |
| -1.6 (-7.0 to 3.8) | **B - TRPZ+LRTI** |  |  |  |  |
| 4.1 (-1.6 to 9.7) | **5.7 (0.9 to 10.5)** | **C - TJA** |  |  |  |
| ***-25.3 (-36.4 to -14.2)*** | ***-23.7 (-33.4 to 14.0)*** | ***-29.4 (-40.2 to -18.6)*** | **D - Arthrodesis** |  |  |
| -10.6 (-22.8 to 1.5) | -9 (-19.9 to 1.9) | ***-14.7 (-26.6 to -2.8)*** | ***14.7 (0.1 to 29.3)*** | **E - TRPZ+LRTI (allo)** |  |
| 1.4 (-9.3 to 12.0) | 3 (-6.1 to 12.1) | -2.7 (-13.0 to 7.6) | ***26.7 (13.4 to 40.0)*** | *12.0 (-2.2 to 26.2)* | **F – Artelon spacer** |

**Suppl. Table 5**

| **A - TRPZ** |  |  |  |  |  |
| --- | --- | --- | --- | --- | --- |
| 0 (-0.3 to 1.2) | **B - TRPZ+LRTI** |  |  |  |  |
| ***0.9 (0.5 to 1.2)*** | ***0.9 (0.6 to 1.2)*** | **C - TJA** |  |  |  |
| -0.4 (-1.7 to 0.8) | -0.4 (-1.6 to 0.8) | ***-1.3 (-2.6 to -0.1)*** | **D - Arthrodesis** |  |  |
| -0.4 (-1.7 to 0.8) | -0.4 (-1.6 to 0.8) | ***-1.3 (-2.6 to -0.1)*** | 0 (-1.7 to 1.7) | **E - TRPZ+LRTI (allo)** |  |
| 0 (-1.1 to 1.1) | 0 (-1.1 to 1.1) | ***-0.9 (-2.0 to 0.2)*** | 0.4 (-1.2 to 2.0) | 0.4 (-1.2 to 2.0) | **F – Artelon spacer** |

**Suppl. Table 6**


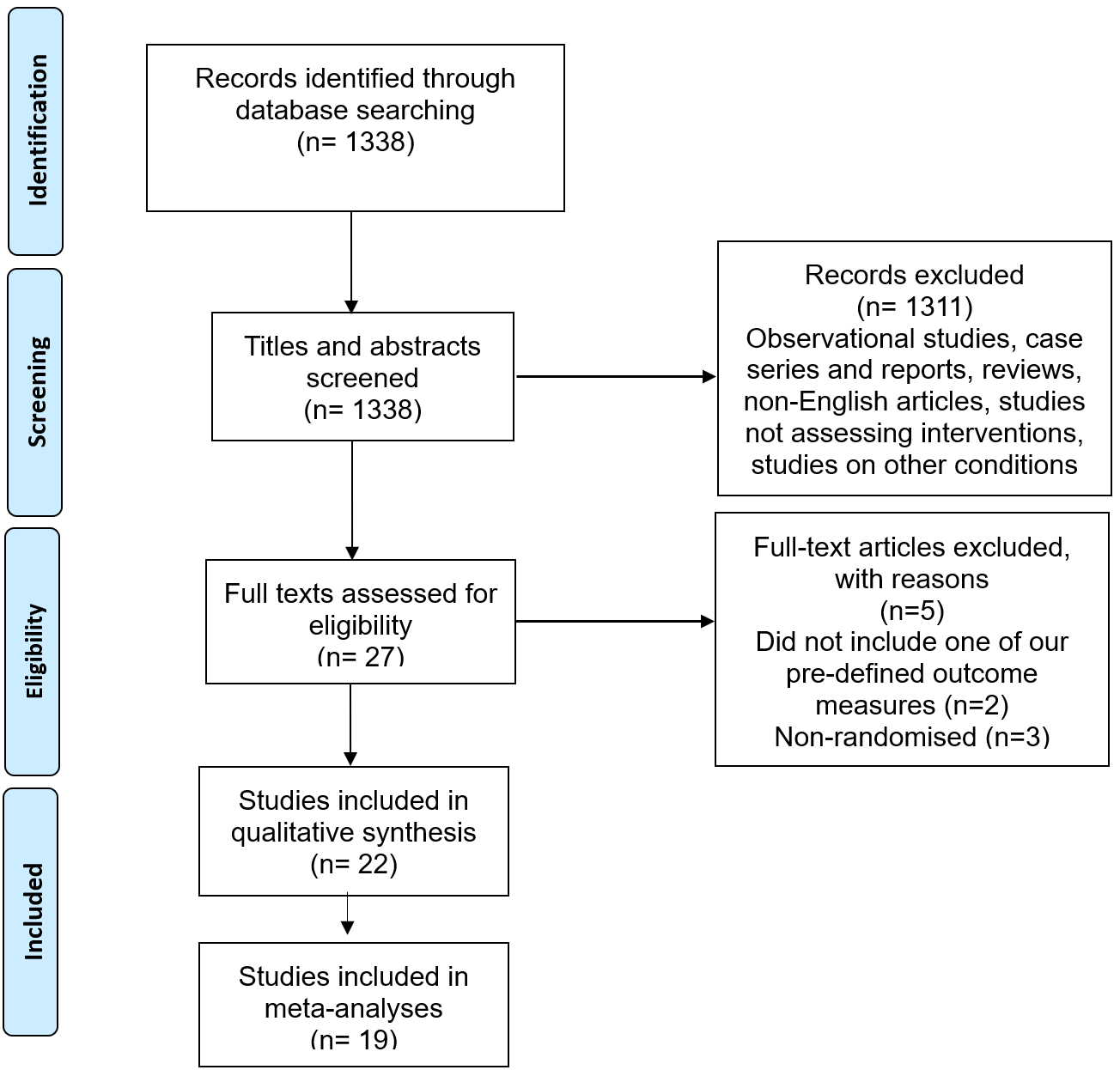
**Figures**

**Suppl. Figure 1**

**Pain**

**
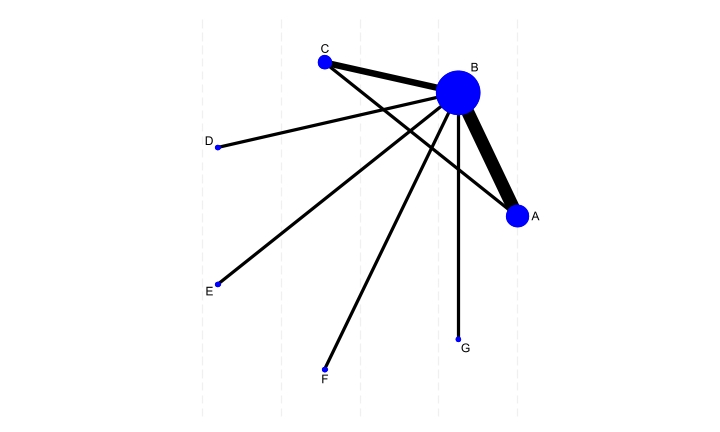
**

**
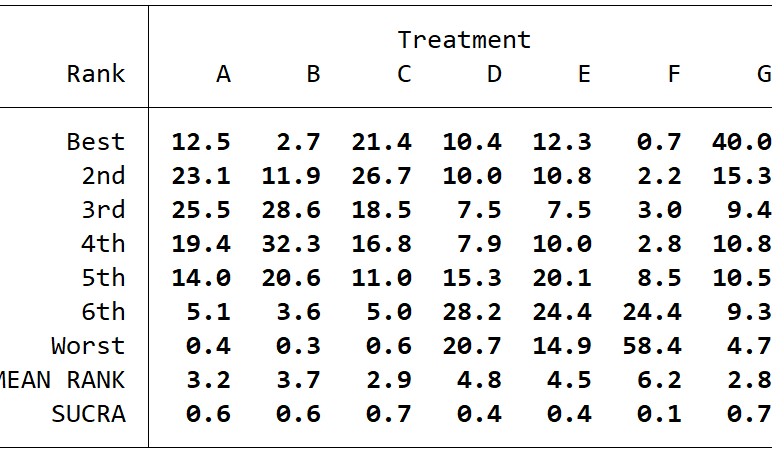
**

**Suppl. Figure 2A**


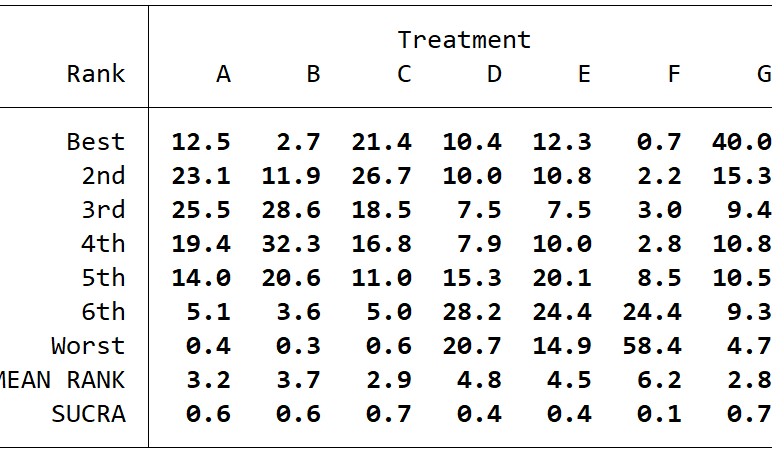


**Suppl. Figure 2B**

**
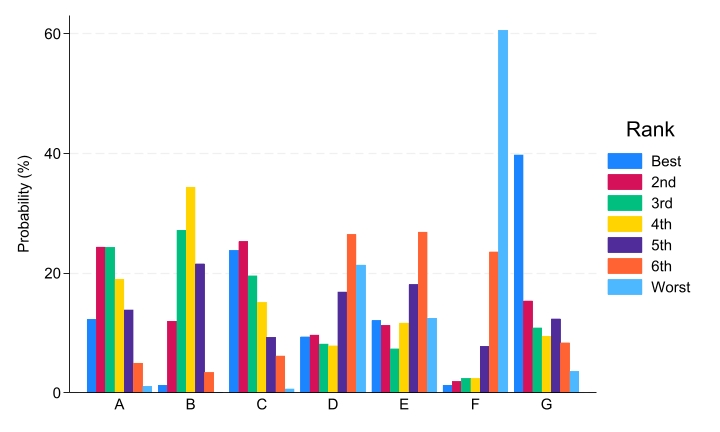
**

**Suppl. Figure 2C**

**Function**

**
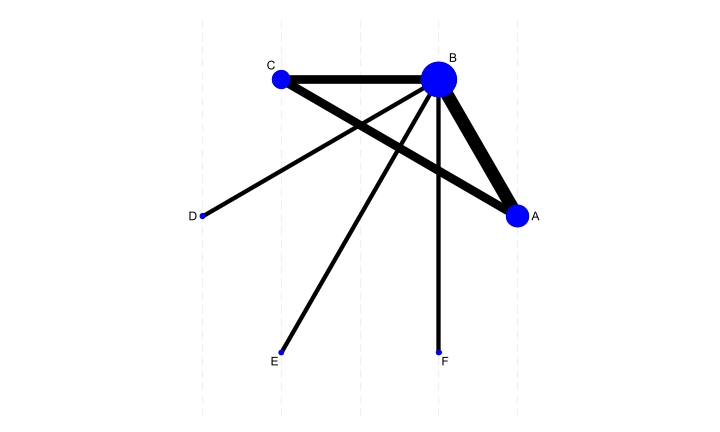
**

**Suppl. Figure 3A**

**
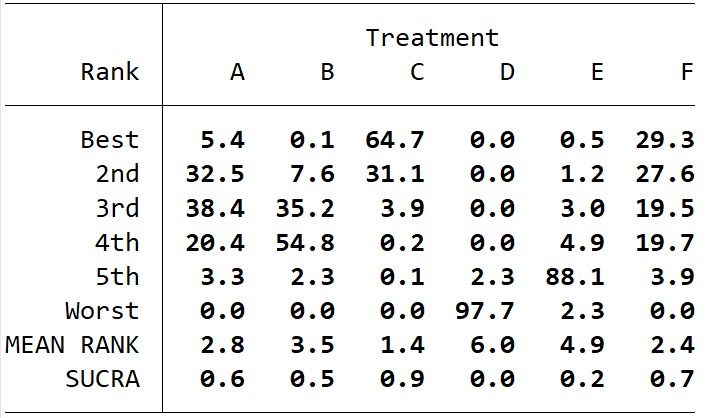
**

**Suppl. Figure 3B**

**
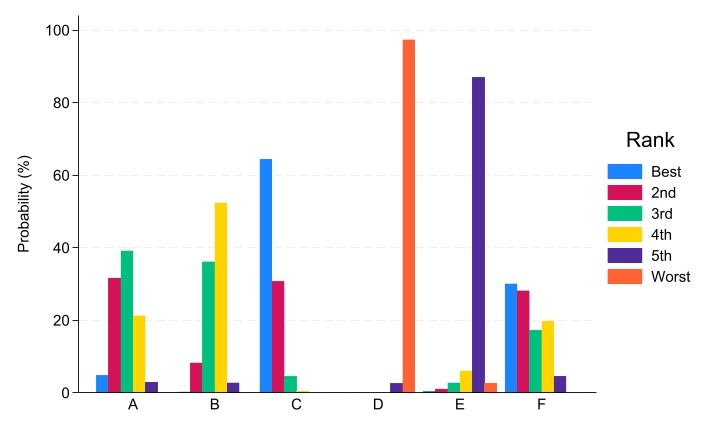
**

**Suppl. Figure 3C**

**Key pinch strength**

**
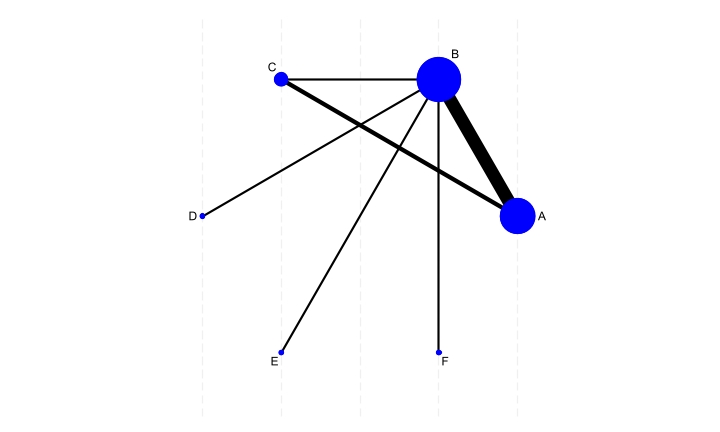
**

**Suppl. Figure 4A**

**
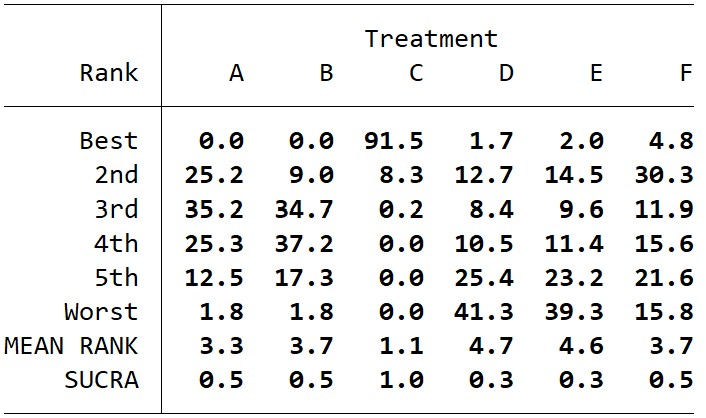
**

**Suppl. Figure 4B**

**
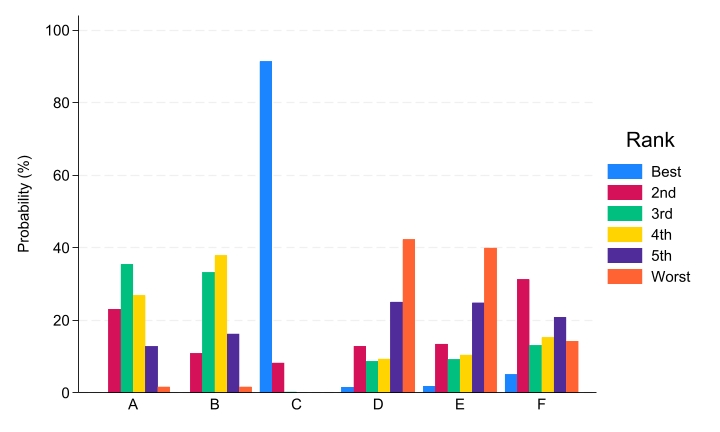
**

**Suppl. Figure 4C**
